# Salinomycin inhibits metastatic colorectal cancer growth and interferes with Wnt/β-catenin signaling in CD133^+^ human colorectal cancer cells

**DOI:** 10.1186/s12885-016-2879-8

**Published:** 2016-11-17

**Authors:** Johannes Klose, Jana Eissele, Claudia Volz, Steffen Schmitt, Alina Ritter, Shen Ying, Thomas Schmidt, Ulrike Heger, Martin Schneider, Alexis Ulrich

**Affiliations:** 1 Department of General, Visceral and Transplantation Surgery, University of Heidelberg, Im Neuenheimer Feld 110, 69120 Heidelberg, Germany; 2 German Cancer Research Center, Im Neuenheimer Feld 280, 69120 Heidelberg, Germany

**Keywords:** Salinomycin, Colorectal cancer, Animal model, Wnt/β-catenin pathway

## Abstract

**Background:**

The polyether antibiotic Salinomycin (Sal) is regarded as an inhibitor of cancer stem cells. Its effectiveness on human colorectal cancer (CRC) cells in vitro has been demonstrated before. The aim of this study was to establish a murine model to investigate the effectiveness of Sal in vivo. Furthermore, we investigated the impact of Sal on Wnt/β-catenin signaling in human CD133^+^ CRC cells.

**Methods:**

The two murine CRC cell lines MC38 and CT26 were used to analyze the impact of Sal on tumor cell proliferation, viability, migration, cell cycle progression and cell death in vitro. For in vivo studies, CT26 cells were injected into syngeneic BALB/c mice to initiate (i) subcutaneous, (ii) orthotopic, or (iii) metastatic CRC growth. Sal was administered daily, 5-Fluoruracil served as a control. For mechanistic studies, the CD133^+^and CD133^-^ subpopulations of human CRC cells were separated by flow cytometry and separately exposed to increasing concentrations of Sal. The impact on Wnt/β-catenin signaling was determined by Western blotting and quantitative PCR.

**Results:**

Sal markedly impaired tumor cell viability, proliferation and migration, and induced necrotic cell death in vitro. CRC growth in vivo was likewise inhibited upon Sal treatment. Interference with Wnt signaling and reduced expression of the Wnt target genes Fibronectin and Lgr5 indicates a novel molecular mechanism, mediating anti-tumoral effects of Sal in CRC.

**Conclusion:**

Sal effectively impairs CRC growth in vivo. Furthermore, Sal acts as an inhibitor of Wnt/β-catenin signaling. Thus, Salinomycin represents a promising candidate for clinical CRC treatment.

**Electronic supplementary material:**

The online version of this article (doi:10.1186/s12885-016-2879-8) contains supplementary material, which is available to authorized users.

## Background

The pro-apoptotic effects of the polyether antibiotic Salinomycin (Sal) in cancer stem cells were first described by Gupta and co-workers [1] and confirmed in succeeding studies in cancer cells of solid and non-solid malignancies (reviewed in [2, 3]). The precise mode of action of Sal is still not completely understood and it is plausible that it differs among the diverse types of cancer cells.

Colorectal cancer (CRC) is the third leading cause of death in the western world [4]. Given that patients’ prognosis in advanced stage of disease is limited and colorectal liver metastases are the most frequent cancer-related death, innovative therapeutic approaches are of utmost importance. The impact of Sal on CRC cells has been already demonstrated [5–7]. In vitro, Sal reduces the CD133^+^ subpopulation of human CRC cells and inhibits epithelial-mesenchymal transition (EMT) [5]. The effect of Sal on CRC has been further explained by induction of autophagy and accumulation of reactive oxygen species [6, 8]. However, there are no data available analyzing the impact of Sal on CRC in vivo.

Hence, the aim of this study was to establish a mouse model to investigate the effectiveness of Sal against CRC growth in vivo. Furthermore, we analyzed the impact of Sal on Wnt signaling in human CD133^+^and CD133^-^ CRC cells. Aberrant Wnt signaling is regarded as crucial for the oncogenesis of CRC [9, 10] and inhibitory effects of Sal on Wnt signaling in other types of cancer but not CRC have been demonstrated before [11].

## Methods

### Cell lines and culture

The murine CRC cell line MC38 [12, 13] was provided by H. Abken (University of Cologne, Germany). CT 26 cells were purchased from the American Type Culture Collection (sub-clone ATTC® CRL2638™) [13]. The human CRC cell line SW620 [14, 15] was obtained from (ATCC); HT29 [15] cells were purchased from the Leibniz Institute DSMZ – German Collection of Microorganisms and Cell Cultures. Cells were cultured in DMEM (Sigma Aldrich) and RPMI 1640 medium (Invitrogen), respectively, supplemented with 10% fetal calf serum, penicillin (50 U/ml) and streptomycin (50 mg/l) at 37°C and 5%CO_2_.

### Chemicals and antibodies

Sal and 5-FU were purchased from Sigma Aldrich. Sal was dissolved in dimethyl sulfoxide (DMSO) for in vitro analysis [16] or in corn oil for in vivo applications [17]. 5-FU was dissolved in phosphate buffered saline (PBS). Stock solutions were stored at -20°C. The CD133 antibody for flow cytometry and cell sorting was purchased from Miltenyi (clone AC133). Antibodies for cleaved (c-) PARP, LRP6 (C47E12), phosphorylated (P-) LRP6 (Ser1490), β-Actin, and β-Tubulin (TU-20) for protein analysis were obtained from Cell Signaling Technology.

### Flow cytometric analysis and cell sorting for CD133^+/-^ cells

Analysis of CD133 positivity was performed according to the manufacturers instructions and as described before [18]. In brief, cells were washed with PBS and stained with a Phycoerythyrin (PE)-conjugated CD133 antibody. Signal enhancement was performed by a two-step FASER procedure (Fluorescence Amplification by Sequential Employment of Reagents). Appropriate isotype antibodies served as control. Cell sorting was performed on a FACS Aria II (Beckton Dickinson). Representative setups before cell sort are depicted in Additional file 1: Figure S1 A + B. The purity of CD133^+^/CD133^-^ cells was analyzed before the experiments were performed (see Additional file 1: Figure S1 C + D). CD133^+^/CD133^-^ cells were maintained in culture for one passage after sorting.

### RNA isolation and real-time PCR

Total RNA from tumor cells and tumor tissues was isolated by an RNA extraction kit (Qiagen). cDNA synthesis and real-time (RT)-PCR were performed using the first strand cDNA synthesis kit (Fermentas) and SYBR Green Master Mix kit (Roche) applying specific primers for human or murine Cyclin D1, Fibronectin, lymphoid enhancer-binding factor 1 (LEF-1) and leucine-rich-repeat-containing G-protein-coupled-receptor 5 (Lgr5). Expression rates of the genes of interest were normalized to the expression of glyceraldehy-3-phosphat-dehydrogenase (GPDH). Primer sequences are listed in Additional file 2: Table S1.

### Western blotting

After drug treatment for 48 h nuclear protein was isolated (Life Technologies). Protein content was determined applying the BCA Protein Assay Kit (Life Technologies). Equal amounts of protein were separated by 10% SDS-PAGE and transferred to PVDF membranes (Milipore). The membranes were incubated overnight with primary antibodies against human LRP6, P-LRP6, and β-catenin. After washing, membranes were incubated and developed with a horseradish peroxidase-conjugated secondary antibody (Life Technologies). β-Tubulin served as internal control. Densitometry quantitative analysis was performed applying Image J software (NIH).

### Proliferation

5 × 10^3^ murine or human CRC cells were cultured in 96-well flat bottom plates. Cells were exposed to increasing concentrations of Sal (1, 2, 5 and 10 μM), to 1 μM 5-FU alone, or to combined Sal and 5-FU for different time periods: either for 24 or 48 h under treatment, or additionally cultured in medium alone for another 24 or 48 h. Cell proliferation was measured using the WST-1 assay, which is based on the cleavage of the tetrazolium salt WST-1 into formazan by healthy cells. After the end of treatment, WST-1 reagent (Roche) was added, followed by further incubation for 4 h. Formazan formation was quantified by measuring the absorbance at 450 nm according to the manufaturer’s instructions.

### Migration

Murine CRC cell migration was further analyzed using an in vitro scratch assay as described before [19]. In brief, 0.5 × 10^6^ MC38 and CT26 cells were cultured in 6-well plates until confluence. A scratch was created in the middle of the monolayer and cells were treated with either Sal or 5-FU alone, or combined Sal and 5-FU. Cell migration was assessed by phase-contrast microscopy (Zeiss, Jena, Germany) and images were captured at the beginning of treatment and after 24 and 48 h. Open wound area was calculated using TScratch software (Swiss Federal Institute of Technology Zurich) as described before [20].

Tumor cell migration was further investigated using transwell-chambers (Cell Biolabs) equipped with an 8 μm pore polycarbonate membrane according to Boyden [21]. 1 × 10^5^ cells were seeded in the upper compartment of the membrane in culture medium without fetal calf serum. The lower compartment of the chamber was filled with culture medium containing 20% fetal calf serum. Cells were cultured in the absence or presence of either Sal or 5-FU alone or combined Sal and 5-FU for 48 h and analyzed immediately or further incubated with fresh culture medium for another 48 h. Afterwards the cells in the upper compartment of the membrane were removed using a cotton swab. Membranes were stained with crystal violet solution, migrated cells on the lower side of the membrane were isolated from the membrane and quantified by measurement of the absorbance at 540 nm according to the manufaturer’s instructions.

### Invasion

Tumor cell invasiveness was analyzed by seeding 1 × 10^5^ cells in Matrigel-coated membranes of transwell-chambers (Cell Biolabs) according to the manufaturer’s instructions and as described before [22]. Tumor cell invasion assay was further performed as described for tumor cell migration assay (see above).

### Cell death

Cells were analyzed for apoptosis, late apoptosis or necrosis induction following exposure to either Sal or 5-FU alone or combined Sal and 5-FU for 24 h applying the AnnexinV apoptosis detection kit (BD Biosciences) according to the manufacturer’s instructions as previously described [16, 23]. AnnexinV positive cells were regarded as apoptotic cells; AnnexinV/PI positive cells were regarded as late apoptotic and PI positive cells were regarded as necrotic cells. Cell death was further evaluated by quantification of DNA fragmentation in cultured MC38 and CT26 cells using the HT Titer TACS Assay Kit (Trevigen) according to the manufacturer’s instructions and as described before [24]. Tumor cell death was analyzed applying the Lactate dehydrogenase (LDH) Cytotoxicity Assay Kit (Thermo Fisher) following the manufacturer’s protocol and as described before [25].

### Cell cycle analysis

Cell cycle analysis was performed after exposure to either Sal, 5-FU or Sal and 5-FU as indicated above for 24 h applying the CellTest Plus Reagent Kit (BD Biosciences) according to the manufaturer’s instructions as described before [23]. Analysis was performed using the Mod Fit LT Software (Verity House Software).

### Animal models and treatment

Animal experiments were carried out in 6–10 week-old BALB/c-mice purchased from Charles River Laboratories. Animals were housed under standard conditions with free access to food and water under constant environmental conditions with a 12-h day-night-cycle. Isoflurane was used for inhalation anesthesia. For a subcutaneous tumor model, 1 × 10^6^ CT26 cells were injected in 50 μl Matrigel (BD Biosciences) into the flank. After 7 days the animals were randomized into 5 treatment groups. The animals were treated daily either with corn oil (control group), 8 mg/kg 5-FU, 4 mg/kg Sal or a combination of 4 mg/kg Sal and 8 mg/kg 5-FU intraperitoneally. Tumor volume was assessed daily during chemotherapy for a total of 14 days.

For the orthotopic CRC model, an abdominal midline incision was performed, the cecum exposed, and 0.5 × 10^6^ CT26 cells (in 50 μl Matrigel) were injected into the cecal wall. Afterwards, the cecum was rinsed with distilled water to kill leaking tumor cells and repositioned into the abdomen. Alternatively, to assess the efficacy of Sal treatment in colorectal liver metastases, after laparotomy the portal vein was exposed and 0.5 × 10^6^ CT26 were injected into the vein. The abdomen was closed using PDS 6-0 running suture. The skin was closed using a 7 mm skin stapler. Mice were checked routinely every day. Explorative laparotomy was performed after 5, 7 and 21 days to evaluate tumor growth during further treatment. Adequate tumour growth was observed after 7 days, and the animals were randomly divided into 5 groups. After end of treatment, mice were sacrificed by cervical dislocation, tumors were harvested, tumor volume assessed and tumor tissue was either snap frozen in liquid nitrogen or embedded in paraffin for further analysis. Based on H&E staining, the metastatic area within the livers was determined morphometrically applying Image J software. 20 pictures from each H&E stained slide (10 slides per animal) were randomly taken and the metastatic lesions marked. Pixels within the marked areas were related to the overall pixel count by Image J software and means ± SD were calculated and expressed as percentage of metastatic area in correlation to the whole liver.

### Terminal desoxynucleotidyl transferase (dUTP) nick end labeling (TUNEL) assay

TUNEL assay for apoptosis detection was performed on tissue slides after removal of paraffin applying the DeadEnd Fluorometric TUNEL system (Promega) according to the manufaturer’s instructions.

### Immunohistochemistry

Paraffin fixed tissue samples were cut into sections of 5 μm and routine hematoxylin and eosin (H&E) staining was performed to evaluate histomorphological features.

### Statistical analysis

Statistical analysis was performed using GraphPadPrism 6. Student’s *t*-test or ANOVA analysis were applied as appropriate. For all in vivo experiments, we gained for an effect (Cohen d) < 2. We supposed an effect d (d = Δ/σ with Δ = relevant difference; σ = standard deviation) of 1.5. Hence, we needed 9 animals per group to confirm this effect α = 0.05 with a power of 80%. We calculated a fault rate of *n* = 3 animals per group. Student’s *t*-test was used for analysis.

Differences were regarded statistically significant with *p* < 0.05 compared to untreated cells which are indicated as “control” below. Results were expressed as mean ± SD of at least three independent experiments.

## Results

### Salinomycin-exposure causes sustained impairment of tumor cell proliferation

**Fig. 1:**
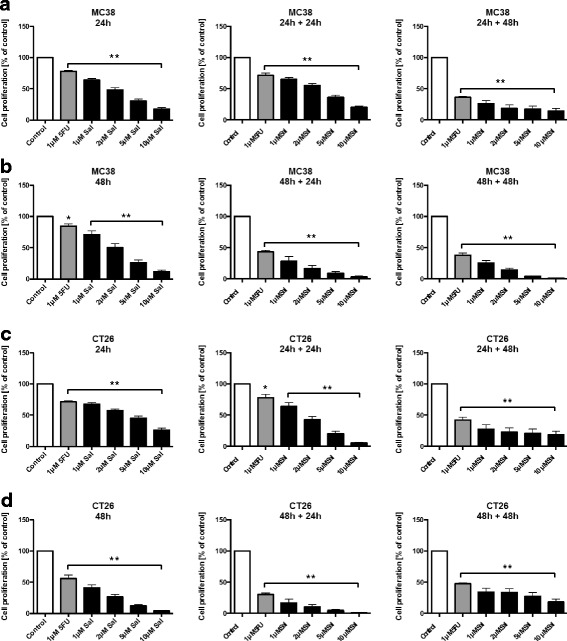
Impaired murine tumor cell proliferation after treatment with Salinomycin. 5 × 10^3^ MC38 (**a** + **b**) and CT26 (**c** + **d**) cells were cultured in microtitre plates in the absence or presence of 1 μM 5-FU, increasing concentrations of Salinomycin (1 μM, 2 μM, 5 μM and 10 μM) and a combination of 5-FU and Salinomycin. Treatment was performed either for 24 h and 48 h or for another 24–48 h with fresh medium. Tumor cell proliferation was performed using the WST-1 assay. Results are displayed as summary of at least 3 independent experiments as mean ± SD; *p < 0.05 and **p < 0.001 compared with control

MC38 and CT26 cells were exposed to increasing concentrations of Sal (1–10 μM) and 5-FU (1 μM) or to a combination of Sal and 5-FU for 24 and 48 h. Alternatively, the cells were further incubated for another 24 or 48 h after the medium was changed. As demonstrated in Fig. 1, Sal-treatment significantly reduced tumor cell proliferation dose-dependently even after lapse of the agent in both MC38 (A + B) and CT26 cells (C + D). Combined treatment with Sal and 5-FU did cause comparable results without an additive synergistic anti-proliferative effect (Additional file 3: Figure S2).

### Reduced tumor cell migration and invasion after Salinomycin-treatment

**Fig. 2:**
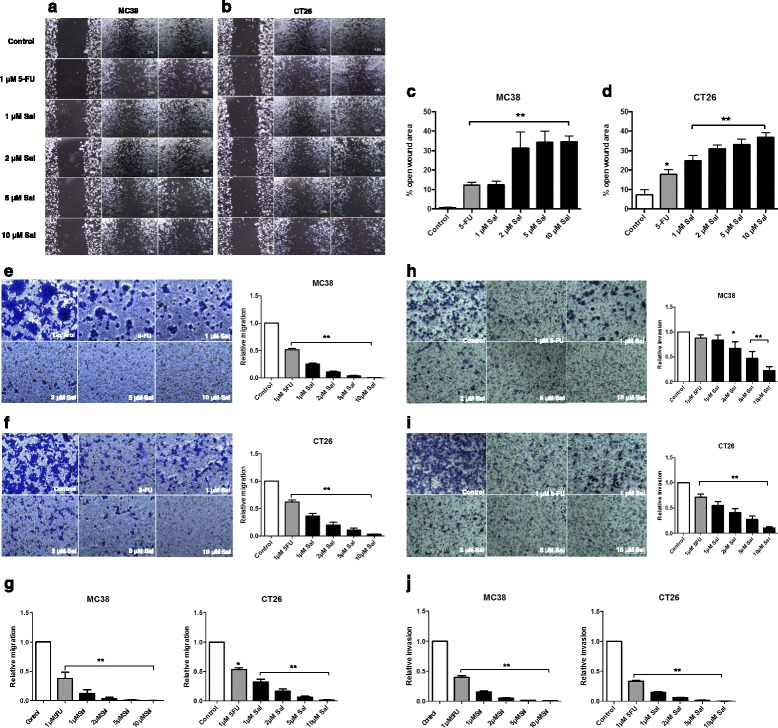
Salinomycin reduces migration and invasion of murine colorectal cancer cells. To visualize tumor cell migration 0.5 × 10^6^ MC38 (**a**) and CT26 (**b**) cells were cultured in 6-well plates until confluence. A scratch was created in the middle of the monolayer and cells were treated with 1 μM 5-FU or increasing concentrations of Sal (1 μM, 2 μM, 5 μM and 10 μM). Cell migration was assessed by phase-contrast microscopy and images were captured at the beginning of treatment and after 24 and 48 h at a magnification of 100. The open wound area after 48 h in cultured MC38 (**c**) and CT26 cells (**d**) was calculated and displayed as a summary of 3 independent experiments as mean ± SD; * *p* < 0.001 compared with control. For transwell-analysis of tumor cell migration 1 × 10^5^ MC38 or CT26 cells were seeded in 6-well plates equipped with a transwell insert and exposed to 1 μM 5-FU, increasing concentrations of Salinomycin (1 μM, 2 μM, 5 μM and 10 μM) and a combination of 5-FU and Salinomycin. After 48 h membranes were stained with crystal violet solution and migrated cells were isolated from the lower side of the membrane and quantified by ELISA reader (**e** + **f**). To assess sustained effects of Salinomycin on tumor cell migration, the cells were treated for 48 h as indicated above and further cultured for another 48 h after lapse of the agent (**g**). Alternatively, MC38 and CT26 were cultured in Matrigel-coated transwell inserts. After 48 h or further incubation for another 48 h with fresh culture medium the number of invasive migrated cells was quantified as described above (**h**-**j**). Results are shown as representative images of stained membranes at a magnification of 100 or as summary of at least 3 independent experiments as mean ± SD; * *p* < 0.05 and ** *p* < 0.001 compared with control

After demonstrating the anti-proliferative effect of Sal on murine CRC cells, we next investigated the impact of Sal on tumor cell migration and invasion. Impaired migration of MC38 and CT26 cells was visualized applying a scratch assay after 24 and 48 h of treatment (Fig. 2 a + b). After 48 h open wound area was clearly increased after treatment with Sal (Fig. 2 c + d). Combined treatment resulted in comparable results (Additional file 4: Figure S3 A-D).

To further quantify the effects of Sal on migration, we used a transwell-assay. Sal significantly decreased trans-membrane migration of MC38 or CT26 cells after treatment for 48 h in a dose-dependent manner (Fig. 2 e + f). Further incubation for another 48 h revealed sustained impairment of tumor cell migration (Fig. 2g). Murine CRC cell invasion through an artificial extracellular matrix using Matrigel-coated membranes was likewise significantly impaired in response to Sal administration (Fig. 2h + i) even after lapse of the agent (Fig. 2j).

Combined treatment with 5-FU and Sal –resulted in similar effects on tumor cell migration and invasion compared to treatment with Sal alone (Additional file 4: Figure S3 E-H).

### Salinomycin induces death of CRC cells

**Fig. 3:**
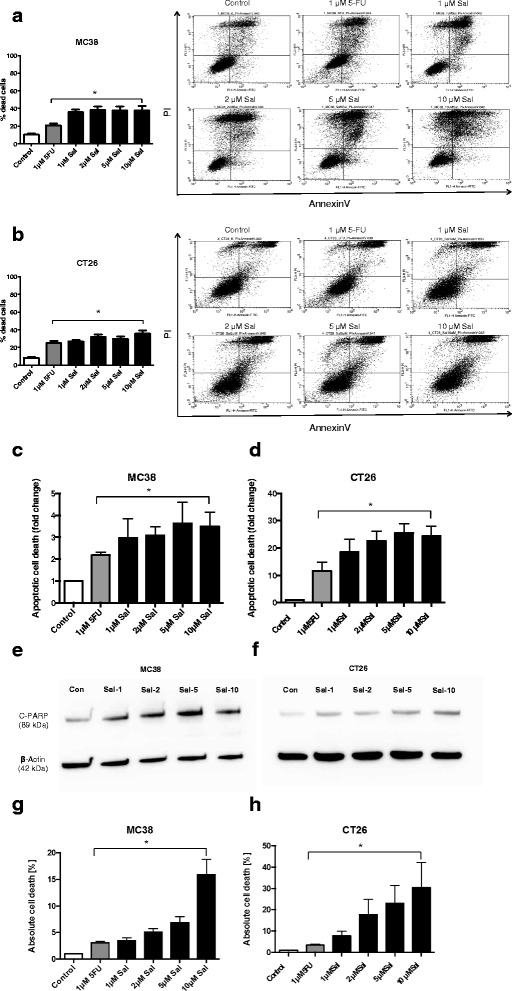
Induction of cell death of murine colorectal cancer cells after treatment with Salinomycin. 0.5 × 10^6^ MC38 (**a**) or CT26 (**b**) cells were seeded in 6-well plates and grown until confluence following exposure to 1 μM 5-FU and increasing concentrations of Salinomycin (1 μM, 2 μM, 5 μM and 10 μM) for 24 h. Detection of cell death was performed using AnnexinV-FITC and PI staining and cells analyzed by flowcytometry. Cell death was further determined by quantification of DNA fragmentation (**c** + **d**), c-PARP production (**e** + **f**) and LDH release assay (**g** + **h**). Results are displayed as representative dot blots or as summary of at least 3 independent experiments; * *p* < 0.05 compared with control

We further analyzed whether Sal induces cell death in MC38 and CT26 cells. As demonstrated in Fig. 3, exposure to Sal was associated with an increased amount of necrotic cells (AnnexinV/PI positive) compared to control or 5-FU-treatment. This effect was dose-dependent in both MC38 (Fig. 3a) and CT26 cells (Fig. 3b). Exposure to Sal likewise resulted in increased DNA fragmentation in MC38 and CT26 cells, respectively (Fig. 3c + d). We further analyzed cleaved poly ADP ribose polymerase (c-PARP) production upon treatment with Sal. Exposure to increasing concentrations of Sal resulted in an increased production of c-PARP (Fig. 3e + f). Cell death was also examined in a LDH release assay. As demonstrated in Fig. 3g and h, treatment with Sal resulted in a dose-dependent increase of LDH release in murine tumor (Fig. 3g) and CT26 cells (Fig. 3h).

The combination of Sal and 5-FU resulted in equivalent effects on tumor cell death (Additional file 4: Figure S3 I + J). We further performed cell cycle analyses of MC38 and CT26 cells after treatment with Sal. The results are summarized in the supplementary results section within the Appendix (see below).

### Salinomycin inhibits growth of murine CRC in vivo by induction of apoptosis

**Fig. 4:**
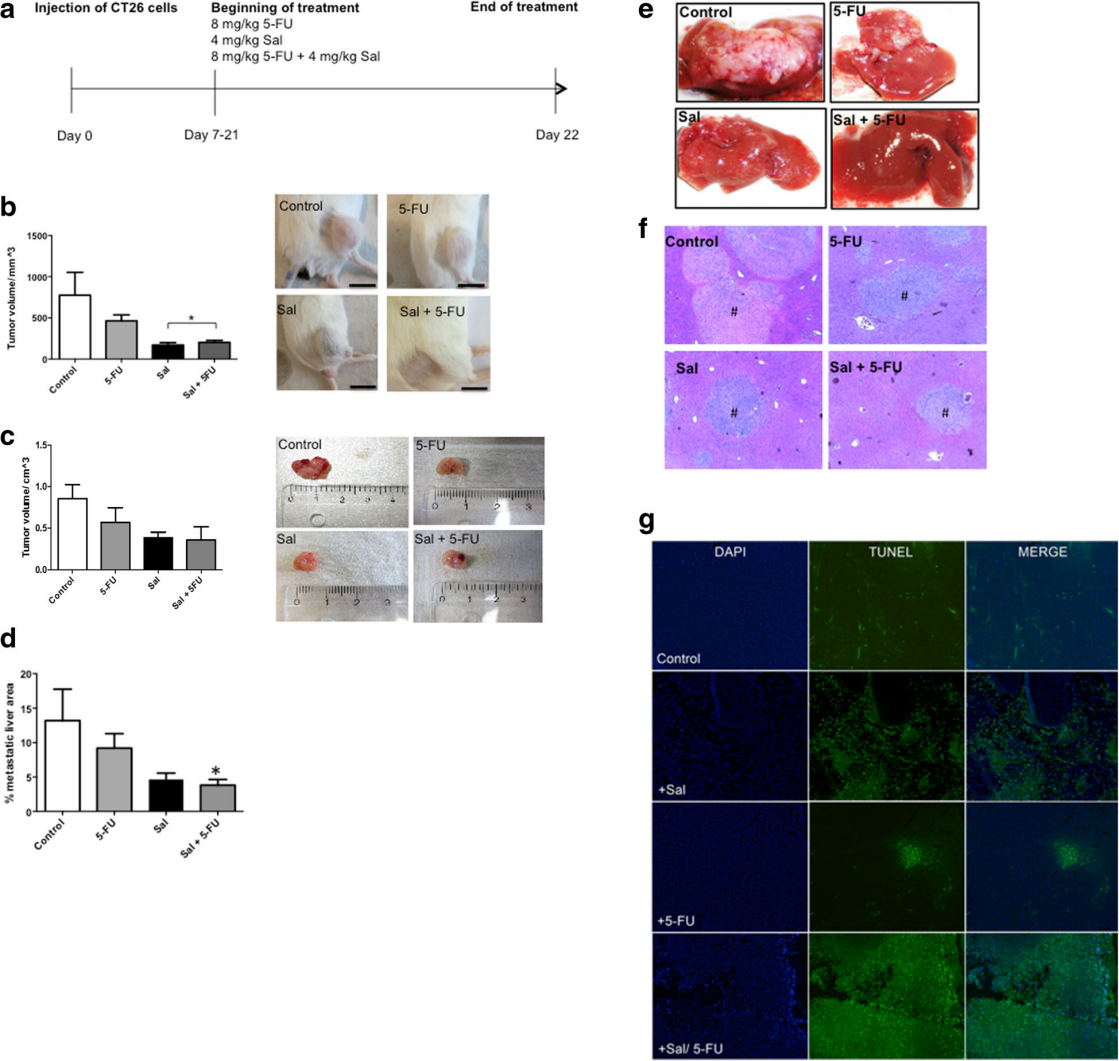
Inhibition of murine colorectal cancer growth in vivo by Salinomycin. Colorectal cancer growth in BALB/c-mice was induced through injection of CT26 cells. Cells were either injected subcutaneously into the flank of the animals, into the wall of the cecum to induce orthotopic tumor growth or into the portal vein to induce metastatic spread. After 1 week, treatment with either 5-FU, Salinomycin or 5-FU and Salinomycin was performed (**a**). **b** After 2 weeks of treatment, Salinomycin significantly inhibited colorectal cancer growth in the subcutaneous tumor model compared to control. Scale bars, 15 mm. **c** Orthotopic colorectal cancer growth was also statistically significant abolished after treatment with Salinomycin for 2 weeks. Results are shown as mean tumor volume ± SD, * *p* < 0.05, ** *p* < 0.001 compared with control. **d**-**f** The distribution of metastatic colorectal cancer spread in the liver of mice (indicated as #) was also markedly reduced after treatment with Salinomycin. Results are shown as representative images of CRC liver spread, H&E stained sections or mean of the percentage of metastatic area ± SD of 9 individual experiments, * *p* < 0.05. **g** TNUEL assay was performed to investigate the amount of apoptotic cells within the explanted murine tumors. After removal of paraffin, slides were stained with TUNEL reagents according to the manufacturers instructions. The displayed result is a representative image of an orthotopic colorectal cancer specimen

To investigate the impact of Sal on CRC growth in vivo, three CRC models (subcutaneous, orthotopic and heterotopic) were applied. Nine animals were initially treated in each group with either daily Sal, 5-FU or a combination of Sal and 5-FU for 2 weeks (see Fig. 4a). Chemotherapy was well tolerated as documented by general health status of the animals and body weight during the treatment. For analysis of the subcutaneous tumor model, we had to remove three animals out of the control group and two animals out of the 5-FU group due to excessive tumor growth. In the orthotopic and liver spread model, all animals were included into the analysis.

In the subcutaneous tumor model, Sal significantly inhibited CRC growth. This growth-inhibiting effect was more pronounced compared to treatment with 5-FU alone. Combined treatment with Sal and 5-FU did not result in an additional growth-inhibiting effect (Fig. 4b).

Sal likewise inhibited orthotopic tumor growth in the cecum of mice. Sal-treated animals presented with smaller tumors compared to 5-FU-treated animals. Combined treatment with Sal and 5-FU did not cause further growth inhibition (Fig. 4c).

A model of colorectal liver spread likewise confirmed the effectiveness of Sal-treatment on CRC in vivo. Sal alone or in combination with 5-Fu resulted in markedly reduction of metastatic liver spread compared to animals treated either with corn oil (control group) or 5-FU alone (Fig. 4d-f).

We further investigated whether Sal induces apoptosis in CRC in vivo, which was performed on paraffin embedded tumor tissue slides by TUNEL assay. As demonstrated in Fig. 4g treatment with Sal alone or in combination with 5-FU resulted in an increased amount of apoptotic tumor area compared to 5-FU alone after 2 weeks of treatment.

### Impact of Salinomycin on human CD133^+^/CD133^-^ CRC cells

After establishing an in vivo model to study the effectiveness of Sal in murine CRC, we sought to determine mechanisms mediating Sal’s anti-tumorigenic effects in human CRC. It is acknowledged that Sal decreases the proportion of CD133^+^ tumor cells, regarded as tumor stem-like cells [5]. Therefore, we analyzed the impact of Sal on human CD133^+^/CD133^-^ CRC cells. To this end, the CD133 expression of seven different human CRC cells (LS174T, HCT116, DLD1, SW480, SW620, CaCo2 and HT29) was analyzed. Only two cell lines (SW620 and HT29) showed a definable proportion of CD133^+^ cells. CD133^+^/CD133^-^ cells were separated by flow cytometry after staining with an appropriate antibody to detect CD133. Afterwards, cells were further cultured separately for one more passage and used for additional experiments.

### Salinomycin impairs proliferation of human CD133^+^/CD133^-^ CRC cells and induces cell death

**Fig. 5:**
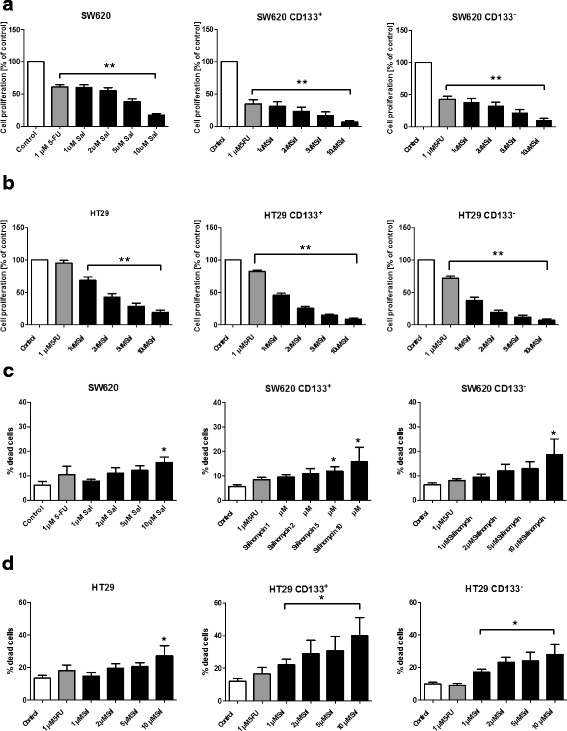
Salinomycin reduces proliferation and induces cell death of human CD133 colorectal cancer cells after treatment with Salinomycin. 5 × 10^3^ SW620 or SW620-CD133^+^/CD133^-^ (**a**) and HT29 or HT29-CD133^+^/CD133^-^ (**b**) cells were cultured in microtitre plates in the absence or presence of 1 μM 5-FU or increasing concentrations of Salinomycin (1, 2, 5 and 10 μM). Treatment was performed for 48 h. Tumor cell proliferation was investigated using the WST-1 assay. Results are displayed as summary or at least 3 independent experiments as mean ± SD; * *p* < 0.05 and ** *p* < 0.001 compared with control. For cell death detection 0.5 × 10^6^ SW620 or SW620-CD133^+^/CD133^-^ (**c**) and HT29 or HT29-CD133^+^/CD133^-^ (**d**) cells were cultured in 6-well plates in the absence or presence of 1 μM 5-FU or increasing concentrations of Salinomycin (1, 2, 5 and 10 μM). AnnexinV/PI staining was performed and cells were analyzed by flowcytometry. Results are displayed as summary or at least 3 independent experiments as mean ± SD; *p < 0.05 compared with control

Unsorted and CD133^+^/CD133^-^ SW620 und HT29 were exposed to increasing concentrations of Sal or 5-FU for 48 h. Cell proliferation was analyzed using the WST-1 assay. As demonstrated in Fig. 5a + b Sal-treatment resulted in a dose-dependent impairment of tumor cell proliferation in unsorted, CD133^+^ and CD133^-^ SW620 and HT29 cells. (Fig. 5a + b).

Next, we investigated whether Sal exerts its anti-proliferative effects on human CD133^+^/CD133^-^ cells by induction of cell death. Therefore, unsorted and CD133^+^/^-^ SW620 and HT29 cells were exposed to Sal or 5-FU as indicated above for 24 h. Cell death was investigated using AnnexinV and PI staining. As depicted in Fig. 5c + d induction of necrotic cell death (AnnexinV/PI double positive cells) by Sal was likewise observed in unsorted and CD133^+^/CD133^-^cells.

### Salinomycin interferes with Wnt-signaling in CD133^+^/CD133^-^ human CRC cells

**Fig. 6:**
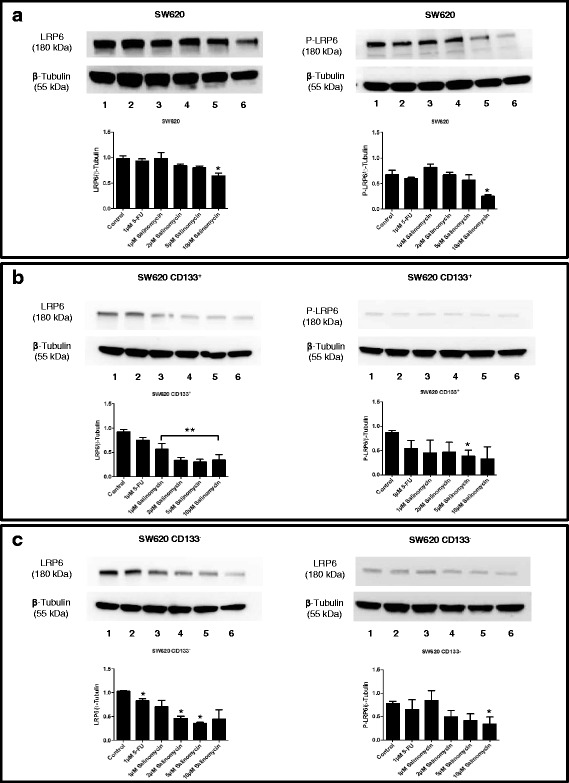
Treatment with Salinomycin interferes with Wnt/β-catenin signaling in human CD133^+^/CD133^-^ colorectal cancer cells. The impact of Salinomycin treatment on the Wnt/β-catenin signaling pathway in SW620 CD133^+^/CD133^-^ cells was investigated. For protein expression analysis 1 × 10^6^ total SW620 (**a**), SW620-CD133^+^ (**b**) or SW620-CD133^-^ (**c**) cells were cultured in 6-well plates in the absence or presence of 1 μM 5-FU or increasing concentrations of Salinomycin (1, 2, 5 and 10 μM). After protein extraction western blotting was performed using specific antibodies against total LRP6 and phosphorylated (P-LRP6). Results are displayed as western blot analysis of LRP6 and P-LRP6 (up) with densitometry quantitative analysis (down). * *p* < 0.05, ** *p* < 0.001 compared to control

To delineate some of the molecular effects of Sal in human CD133^+^and CD133^-^ CRC cells, we analyzed Wnt signaling after Sal-treatment. As demonstrated in Fig. 6, treatment with Sal interferes with Wnt signaling cascade in both unsorted and CD133^+^/CD133^-^ SW620 cells. Sal-treatment resulted in reduced LRP6 protein levels and inhibited LRP6-phosphorylation (Fig. 6). When analyzing unsorted and CD133^+^/CD133^-^ HT29 cells, similar effects of Sal on Wnt-signaling were found (Additional file 5: Figure S4).

**Fig. 7:**
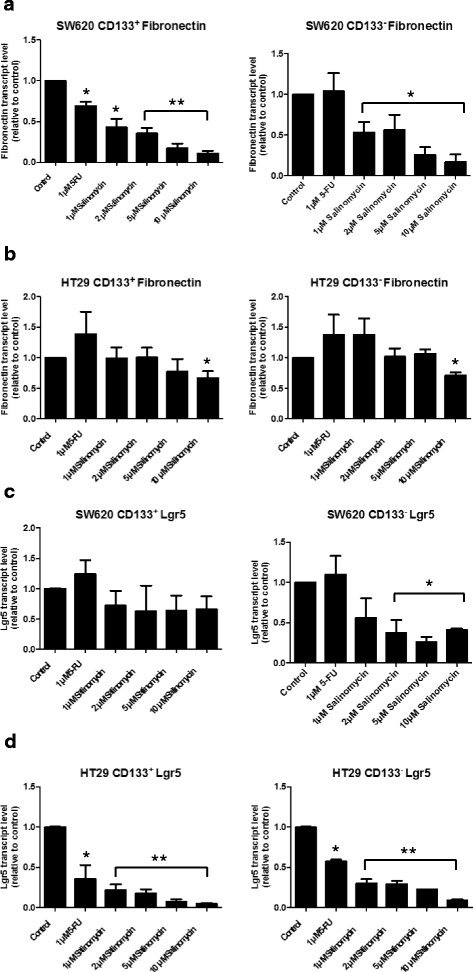
Salinomycin down-regulates the mRNA expression of Fibronectin and Lgr5 in human CD133^+^/CD133^-^ colorectal cancer cells. The mRNA expression levels of Fibronectin (**a** + **b**) and Lr5 (**c** + **d**) in CD133^+^/CD133^-^ SW620 and CD133^+^/CD133^-^ HT29 cells were measured by quantitative PCR after treatment with1 μM 5-FU or increasing concentrations of Salinomycin (1, 2, 5 and 10 μM). * *p* < 0.05, ** *p* < 0.001 compared to control

Next, we analyzed if the inhibitory effects of Sal on Wnt signaling cascade also result in alterations of mRNA expression of target genes of the Wnt/β-catenin pathway. Therefore, we analyzed the mRNA expression levels of Cyclin D1, LEF-1, Fibronectin and Lgr5 using quantitative PCR after 24 h of treatment. In both CD133^+^ and CD133^-^ SW620 cells, we observed a decreased expression of Fibronectin in a dose-dependent manner after exposure to Sal (see Fig. 7a). Reduced expression of Fibronectin was also observed in CD133^+^ SW620 cells after exposure to 5-FU. Likewise, a reduced mRNA expression of Fibronectin was observed in CD133^+^and CD133^-^ HT29 cells after treatment with Sal (Fig. 7b).

Regarding the expression of Lgr5 mRNA, we also observed a decreased expression in CD133^+^/CD133^-^ SW620 and HT29 cells The dose-dependent decreased expression of Lgr5 mRNA was more pronounced in CD133^+^ and CD133^-^ HT29 cells compared to CD133^+^/CD133^-^ SW620 cells (see Fig. 7c + d). There were no consistent changes in the mRNA expression of Cyclin D1 and LEF-1 (see Additional file 6: Figure S5).

## Discussion

In this study we demonstrate that Sal exerts a growth-inhibiting effect on murine CRC in vivo. Furthermore, we provide evidence that the pro-apoptotic effect of Sal on human CD133^+^ CRC cells is associated with impaired Wnt signaling and reduced expression of Wnt target genes.

The potential of Sal to treat CRC has been demonstrated in vitro before [5, 6, 8]. However, the evidence for the effectiveness of Sal in CRC in vivo is still missing. We therefore established a murine model to investigate the impact of Sal on CRC in vivo. First, we demonstrated that Sal exerts its pro-apoptotic effect in two murine CRC cell lines in a dose-dependent manner. This is accompanied by inhibition of tumor cell migration and invasion. Interestingly, in contrast to other observations before, an additive synergistic effect of Sal and 5-FU in vitro was not observed [26–28].

The obtained data providing the effectiveness of Sal in murine CRC in vitro encouraged us to establish an in vivo-model for CRC. First, we induced subcutaneous CRC growth in BALB/c-mice. Treatment with Sal resulted in inhibition of tumor growth compared to control or 5-FU-treatment. Orthotopic tumor growth in the cecum of the mice was likewise abolished after treatment with Sal. Additionally, Sal inhibited colorectal spread in the liver of mice. Treatment with Sal alone or in combination with 5-FU was superior combined to 5-FU alone. Given that CRC liver metastasis are the leading cause for CRC-related death [4] this observation promises an important clinical impact. The effectiveness of Sal in vivo has been described before predominantly in subcutaneous tumor models [1, 7, 17, 26, 29–31]. Our data demonstrate the effectiveness of Sal in clinical relevant CRC models for the first time in a convincing extent.

Next, we gained to dissect a molecular mechanism of Sal. Based on the observation that Sal reduces the CD133^+^ and therewith stem-like cell signature subpopulations of human CRC cells [5], we investigated if the mode of action of Sal in CD133^+^ and CD133^-^ cells varies. We focused on Wnt signaling in human CD133^+^ and CD133^-^ CRC cell lines given that aberrations in Wnt signaling cascade and its impact on CRC development are well known [9, 10, 32–34]. Additionally, the inhibitory effect of Sal on Wnt signaling has been described in other tumor entities before, including leukemia, breast, pancreatic, prostate, and lung cancer cells [11, 26, 35–38]. Hence, the hypothesis that Salinomycin acts as an inhibitor of Wnt signaling is not new. On the other hand, there is no data available indicating that Sal has an influence on Wnt signaling in CRC. The following findings led us to the conclusion that interference with the Wnt/β-catenin pathway might be responsible for the pro-apoptotic effect of Sal in human CRC cells.

First, we exposed SW620 and HT29 and the CD133^+^/^-^ subpopulations of each cell line separately to Sal and confirmed the toxic effect on all CRC cell subpopulations. Interestingly, the effect of Sal on the CD133^+^ and CD133^-^ subpopulations did not differ regarding impairment of proliferation and induction of apoptosis.

Next, we observed blocking of LRP6-phosphorylation by Sal and reduced LRP6 levels in both CD133^+^ and CD133^-^ cells. The phosphorylation of the LRP6 co-receptor is crucial for the activation of the Wnt/β-catenin pathway [11, 36] and in aberrant Wnt signaling-associated carcinogenesis [39] of CRC. Reduced phosphorylation of LRP6 after exposure to Sal was first described in chronic lymphocytic leukemia cells [36]. Our results further confirm the data obtained by Lu and co-workers in another study on Sal and Wnt signaling. They observed suppression of LRP6-expression in prostate and breast cancer cells after Sal-treatment [37]. Additionally, they also described reduced expression of the Wnt target genes Cyclin D1 and Survivin [37]. We further analyzed the expression profiles of selected Wnt target genes. Fibronectin and Lgr5 mRNA expression were both down-regulated upon treatment with Sal in SW620 and HT29 cells. The importance of Fibronectin and its spliced variant extra domain A (EDA) has been characterized as essential for the phenotype and the tumorigenic properties of CD133^+^/CD44^+^ CRC cells [40]. Furthermore, a correlation between Fibronectin EDA-level and stage of disease and chemoresistance of CRC patients was reported [40]. Given that silencing of EDA resulted in downregulation of Wnt/β-catenin signaling and the inhibitory effects of Sal on Fibronectin expression in our study, Sal might be regarded as an inhibitor of the regulatory Fibronectin/Wnt/β-catenin signaling loop in human CRC cells [41].

Lgr5 is a Wnt target gene that acts as a receptor for the Wnt agonist R spondin [42, 43]. The implications of Lgr5 expression and its tumorigenic activity in human CRC were described before indicating a crucial role in stem-like cells of CRC [44, 45]. Silencing of Lgr5 resulted in reduced proliferation, migration and colony formation and decreased tumorigenic activity in vivo [44]. Zhou et al. reported that Sal is able to overcome Cisplatin-resistance in human CRC cells displaying stem-like signatures, including increased Lgr5 expression [8]. To the best of our knowledge, our study is the first to show that treatment with Sal directly inhibits Lgr5 expression in human CRC cells. Having in mind the impaired tumorigenicity after silencing of Lgr5, the toxic effect of Sal in CRC cells are plausible.

## Conclusions

In summary, our results demonstrate that Sal remains a candidate for a potential pre-clinical application for CRC-treatment. Interference with the Wnt/β-catenin signaling cascade and consecutive reduced expression of the Wnt target genes Fibronectin and Lgr5 might represent a novel molecular mechanism of Sal in CRC. Further studies in primary human CRC specimens are necessary to delineate the implication for Sal in targeted therapies in CRC in the future.

## Appendix

### Supplementary results

#### Salinomycin influences the cell cycle of murine CRC cells

MC38 and CT26 cells were exposed to Sal or 5-FU for 24 hours. Cell cycle analysis was based on propidium iodide staining and analyzed by using the Mod Fit LT software. As demonstrated in Supplementary Figure S2, cell cycle analysis revealed equal proportions of untreated MC38 and CT26 cells in the G1-, G2- and S-phases of the cell cycle. Treatment of MC38 cells with 5-FU resulted in a decreased number of cells in the G1 and G2 phases, and an increased number of cells in the S phase. After exposure to high concentrations of Sal (5 and 10 µM), MC38 cells tended to accumulate in the G2 phase and a decreased amount in the G1 phase, while the number of cells in the S phase was rather stable compared to control (Additional file 7: Figure S6 A). In contrast, treatment of CT26 cells with 5-FU resulted an increased number of cells in the G1 phase, whereas the proportion of cells in the G2 and S phases was decreased. After exposure to Sal, CT26 cells reacted with an accumulation in the G1 phase, while the number of cells in the S phase decreased and the proportion of cells in the G2 phase was stable (Additional file 7: Figure S6 B). Combined treatment with Sal and 5-FU did result in similar effects on the cell cycle (data not shown).

## Additional files

Additional file 1: Figure S1. Sorting of CD133^+^ and CD133^-^ human colorectal cancer cells. SW620 and HT29 cells were stained with a Phycoerythyrin-conjugated CD133 antibody. Signal enhancement was performed by a two-step FASER procedure. (A + B) Representative setups before sort of CD133^+^ and CD133^-^ SW620 and HT29 cells. (C + D) The purity of CD133^+^/CD133^-^ cells was analyzed before the experiments were performed. (TIFF 2702 kb)
Additional file 2: Table S1. Sense and anti-sense primer sequences of Wnt target genes and housekeeping gene used for quantitative PCR analysis. (TIFF 2702 kb)
Additional file 3: Figure S2. The impact of combined treatment with Salinomycin and 5-FU on murine CRC cell proliferation. The effect of combined treatment with Salinomycin and 5-FU on tumor cell proliferation was performed using the WST-1 assay. Therefore, 5 × 10^3^ MC38 (A + B) and CT26 (C + D) cells were cultured in microtitre plates in the absence or presence of Salinomycin (1 μM, 2 μM, 5 μM and 10 μM) and 1 μM 5-FU. Treatment was performed either for 24 h and 48 h or for another 24–48 h with fresh medium. Results are displayed as summary of at least 3 independent experiments as mean ± SD; * *p* < 0.05 and ** *p* < 0.001 compared with control. (TIFF 2702 kb)
Additional file 4: Figure S3. Combined treatment with Salinomycin and 5-FU reduces migration and invasion of murine CRC cells and induces necrotic cell death. To visualize tumor cell migration 0.5 × 10^6^ MC38 (A) and CT26 (B) cells were cultured in 6-well plates until confluence. A scratch was created in the middle of the monolayer and cells were treated with Salinomycin (1 μM, 2 μM, 5 μM and 10 μM) and 1 μM 5-FU. Cell migration was assessed by phase-contrast microscopy and images were captured at the beginning of treatment and after 24 and 48 h at a magnification of 100. The open wound area after 48 h in cultured MC38 (C) and CT26 cells (D) was calculated and displayed as a summary of 3 independent experiments as mean ± SD; ** *p* < 0.001 compared with control. For transwell-analysis of tumor cell migration 1 × 10^5^ MC38 or CT26 cells were seeded in 6-well plates equipped with a transwell insert and exposed to Salinomycin (1 μM, 2 μM, 5 μM and 10 μM) and 1 μM 5-FU. After 48 h membranes were stained with crystal violet solution and migrated cells were isolated from the lower side of the membrane and quantified by ELISA reader (E + F). Alternatively, MC38 and CT26 were cultured in Matrigel-coated transwell inserts. After 48 h or further incubation for another 48 h with fresh culture medium the number of invasive migrated cells was quantified as described above (G + H). Results are shown as representative images of stained membranes at a magnification of 100 or as summary of at least 3 independent experiments as mean ± SD; * *p* < 0.05 and ** *p* < 0.001 compared with control. For detection of cell death 0.5 × 10^6^ MC38 (I) or CT26 (J) cells were seeded in 6-well plates and grown until confluence following exposure to Salinomycin (1, 2, 5 and 10 μM) and 1 μM 5-FU for 24 h. Detection of cell death was performed using AnnexinV-FITC and PI staining and cells analyzed by flowcytometry. Results are displayed as representative dot blots or as summary of at least 3 independent experiments; ** *p* < 0.001 compared with control. (ZIP 370 kb)
Additional file 5: Figure S4. Salinomycin interferes with Wnt signaling in HT20 CD133^+/-^ cells. The impact of Salinomycin treatment on the Wnt/β-catenin signaling pathway in HT29 CD133^+^/CD233^-^ cells was investigated. For protein expression analysis 1 × 10^6^ total HT29 (A), HT29-CD133^+^ (B) or HT29-CD133^-^ (C) cells were cultured in 6-well plates in the absence or presence of 1 μM 5-FU or increasing concentrations of Salinomycin (1 μM, 2 μM, 5 μM and 10 μM). After protein extraction western blotting was performed using specific antibodies against total LRP6 and phosphorylated (P-LRP6). (TIFF 2702 kb)
Additional file 6: Figure S5. mRNA expression pattern of Cyclin D1 and LEF1 after treatment with Salinomycin. The mRNA expression levels of Cyclin D1 (A + B) and LEF1 (C + D) in CD133^+^CD133^-^ SW620 and CD133^+^/CD133^-^ HT29 cells were measured by quantitative PCR after treatment with1 μM 5-FU or increasing concentrations of Salinomycin (1 μM, 2 μM, 5 μM and 10 μM). (TIFF 2702 kb)
Additional file 7: Figure S6. Salinomycin influences the cell cycle of murine CRC cells. For cell cycle analysis 1 × 10^3^ MC38 or CT26 cells were seeded in 6-well plates and grown until confluence and then further cultured with medium, 1 μM FU and 5 or 10 μM Salinomycin for 24 h. After the end of treatment, cells were stained with PI and analyzed by flowcytometry. Sal-treatment resulted in a accumulation in the G2 phase in MC38 cells and accumulation in the G1 phase in CT26 cells, respectively. Results are summarized of at least 3 independent experiments. (TIFF 2702 kb)

## Abbreviations

5-FU: 5-Fluoruracil
CRC: Colorectal cancer
LEF1: Lymphoid enhancer-binding factor 1
Lgr5: Leucine-rich-repeat-containing G-protein-coupled-receptor 5
Sal: Salinomycin
Wnt: Wingless-type

## References

[1] Gupta PB, Onder TT, Jiang G, Tao K, Kuperwasser C, Weinberg RA, Lander ES (2009). Identification of selective inhibitors of cancer stem cells by high-throughput screening. Cell.

[2] Naujokat C, Fuchs D, Opelz G (2010). Salinomycin in cancer: A new mission for an old agent. Mol Med Rep.

[3] Naujokat C, Steinhart R (2012). Salinomycin as a drug for targeting human cancer stem cells. J biomed biotechnol.

[4] Weitz J, Koch M, Debus J, Hohler T, Galle PR, Buchler MW (2005). Colorectal cancer. Lancet (London, England).

[5] Dong TT, Zhou HM, Wang LL, Feng B, Lv B, Zheng MH (2011). Salinomycin selectively targets ‘CD133 + ’ cell subpopulations and decreases malignant traits in colorectal cancer lines. Ann Surg Oncol.

[6] Verdoodt B, Vogt M, Schmitz I, Liffers ST, Tannapfel A, Mirmohammadsadegh A (2012). Salinomycin induces autophagy in colon and breast cancer cells with concomitant generation of reactive oxygen species. PLoS One.

[7] Zhang B, Wang X, Cai F, Chen W, Loesch U, Zhong XY (2013). Antitumor properties of salinomycin on cisplatin-resistant human ovarian cancer cells in vitro and in vivo: involvement of p38 MAPK activation. Oncol Rep.

[8] Zhou J, Li P, Xue X, He S, Kuang Y, Zhao H, Chen S, Zhi Q, Guo X (2013). Salinomycin induces apoptosis in cisplatin-resistant colorectal cancer cells by accumulation of reactive oxygen species. Toxicol Lett.

[9] Kinzler KW, Vogelstein B (1996). Lessons from hereditary colorectal cancer. Cell.

[10] Sancho E, Batlle E, Clevers H (2004). Signaling pathways in intestinal development and cancer. Annu Rev Cell Dev Biol.

[11] Lu D, Carson DA (2011). Inhibition of Wnt signaling and cancer stem cells. Oncotarget.

[12] Brattain MG, Strobel-Stevens J, Fine D, Webb M, Sarrif AM (1980). Establishment of mouse colonic carcinoma cell lines with different metastatic properties. Cancer Res.

[13] Corbett TH, Griswold DP, Roberts BJ, Peckham JC, Schabel FM (1975). Tumor induction relationships in development of transplantable cancers of the colon in mice for chemotherapy assays, with a note on carcinogen structure. Cancer Res.

[14] McNutt NS, Mak LL, Kim YS (1981). Comparison of cell peripheries in the human colonic adenocarcinoma cell lines SW480 and SW620 grown in floating chamber culture, cover slip culture, athymic (nude) mice, and BALB/c mice. Lab Invest.

[15] von Kleist S, Chany E, Burtin P, King M, Fogh J (1975). Immunohistology of the antigenic pattern of a continuous cell line from a human colon tumor. J Natl Cancer Inst.

[16] Klose J, Stankov MV, Kleine M, Ramackers W, Panayotova-Dimitrova D, Jager MD, Klempnauer J, Winkler M, Bektas H, Behrens GM (2014). Inhibition of autophagic flux by salinomycin results in anti-cancer effect in hepatocellular carcinoma cells. PLoS One.

[17] Schenk M, Aykut B, Teske C, Giese NA, Weitz J, Welsch T (2015). Salinomycin inhibits growth of pancreatic cancer and cancer cell migration by disruption of actin stress fiber integrity. Cancer Lett.

[18] Elsaba TM, Martinez-Pomares L, Robins AR, Crook S, Seth R, Jackson D, McCart A, Silver AR, Tomlinson IP, Ilyas M (2010). The stem cell marker CD133 associates with enhanced colony formation and cell motility in colorectal cancer. PLoS One.

[19] Liang CC, Park AY, Guan JL (2007). In vitro scratch assay: a convenient and inexpensive method for analysis of cell migration in vitro. Nat Protoc.

[20] Geback T, Schulz MM, Koumoutsakos P, Detmar M (2009). TScratch: a novel and simple software tool for automated analysis of monolayer wound healing assays. BioTechniques.

[21] Boyden S (1962). The chemotactic effect of mixtures of antibody and antigen on polymorphonuclear leucocytes. J Exp Med.

[22] Repesh LA (1989). A new in vitro assay for quantitating tumor cell invasion. Invasion Metastasis.

[23] Lieke T, Ramackers W, Bergmann S, Klempnauer J, Winkler M, Klose J (2012). Impact of Salinomycin on human cholangiocarcinoma: induction of apoptosis and impairment of tumor cell proliferation in vitro. BMC Cancer.

[24] Sokalska A, Wong DH, Cress A, Piotrowski PC, Rzepczynska I, Villanueva J, Duleba AJ (2010). Simvastatin induces apoptosis and alters cytoskeleton in endometrial stromal cells. J Clin Endocrinol Metab.

[25] Grubor-Bauk B, Yu W, Wijesundara D, Gummow J, Garrod T, Brennan AJ, Voskoboinik I, Gowans EJ (2016). Intradermal delivery of DNA encoding HCV NS3 and perforin elicits robust cell-mediated immunity in mice and pigs. Gene Ther.

[26] Wang F, Dai W, Wang Y, Shen M, Chen K, Cheng P, Zhang Y, Wang C, Li J, Zheng Y (2014). The synergistic in vitro and in vivo antitumor effect of combination therapy with salinomycin and 5-fluorouracil against hepatocellular carcinoma. PLoS One.

[27] Zhang GN, Liang Y, Zhou LJ, Chen SP, Chen G, Zhang TP, Kang T, Zhao YP (2011). Combination of salinomycin and gemcitabine eliminates pancreatic cancer cells. Cancer Lett.

[28] Zhou Y, Liang C, Xue F, Chen W, Zhi X, Feng X, Bai X, Liang T (2015). Salinomycin decreases doxorubicin resistance in hepatocellular carcinoma cells by inhibiting the beta-catenin/TCF complex association via FOXO3a activation. Oncotarget.

[29] Larzabal L, El-Nikhely N, Redrado M, Seeger W, Savai R, Calvo A (2013). Differential effects of drugs targeting cancer stem cell (CSC) and non-CSC populations on lung primary tumors and metastasis. PLoS One.

[30] Mao J, Fan S, Ma W, Fan P, Wang B, Zhang J, Wang H, Tang B, Zhang Q, Yu X (2014). Roles of Wnt/beta-catenin signaling in the gastric cancer stem cells proliferation and salinomycin treatment. Cell Death Dis.

[31] Wang F, He L, Dai WQ, Xu YP, Wu D, Lin CL, Wu SM, Cheng P, Zhang Y, Shen M (2012). Salinomycin inhibits proliferation and induces apoptosis of human hepatocellular carcinoma cells in vitro and in vivo. PLoS One.

[32] Clevers H (2006). Wnt/beta-catenin signaling in development and disease. Cell.

[33] Gregorieff A, Clevers H (2005). Wnt signaling in the intestinal epithelium: from endoderm to cancer. Genes Dev.

[34] Reya T, Clevers H (2005). Wnt signalling in stem cells and cancer. Nature.

[35] He L, Wang F, Dai WQ, Wu D, Lin CL, Wu SM, Cheng P, Zhang Y, Shen M, Wang CF (2013). Mechanism of action of salinomycin on growth and migration in pancreatic cancer cell lines. Pancreatol.

[36] Lu D, Choi MY, Yu J, Castro JE, Kipps TJ, Carson DA (2011). Salinomycin inhibits Wnt signaling and selectively induces apoptosis in chronic lymphocytic leukemia cells. Proc Natl Acad Sci U S A.

[37] Lu W, Li Y (2014). Salinomycin suppresses LRP6 expression and inhibits both Wnt/beta-catenin and mTORC1 signaling in breast and prostate cancer cells. J Cell Biochem.

[38] Zhang X, Lou Y, Zheng X, Wang H, Sun J, Dong Q, Han B (2015). Wnt blockers inhibit the proliferation of lung cancer stem cells. Drug Des Devel Ther.

[39] Lemieux E, Cagnol S, Beaudry K, Carrier J, Rivard N: Oncogenic KRAS signalling promotes the Wnt/beta-catenin pathway through LRP6 in colorectal cancer. Oncogene. 2015;34(38):4914-27. doi:10.1038/onc.2014.416. Epub 2014 Dec 15. 10.1038/onc.2014.416 PMC4687460 25500543

[40] Ou J, Deng J, Wei X, Xie G, Zhou R, Yu L, Liang H (2013). Fibronectin extra domain A (EDA) sustains CD133(+)/CD44(+) subpopulation of colorectal cancer cells. Stem Cell Res.

[41] Gradl D, Kuhl M, Wedlich D (1999). The Wnt/Wg signal transducer beta-catenin controls fibronectin expression. Mol Cell Biol.

[42] Carmon KS, Gong X, Lin Q, Thomas A, Liu Q (2011). R-spondins function as ligands of the orphan receptors LGR4 and LGR5 to regulate Wnt/beta-catenin signaling. Proc Natl Acad Sci U S A.

[43] de Lau W, Barker N, Low TY, Koo BK, Li VS, Teunissen H, Kujala P, Haegebarth A, Peters PJ, van de Wetering M (2011). Lgr5 homologues associate with Wnt receptors and mediate R-spondin signalling. Nature.

[44] Hirsch D, Barker N, McNeil N, Hu Y, Camps J, McKinnon K, Clevers H, Ried T, Gaiser T (2014). LGR5 positivity defines stem-like cells in colorectal cancer. Carcinogenesis.

[45] Merlos-Suarez A, Barriga FM, Jung P, Iglesias M, Cespedes MV, Rossell D, Sevillano M, Hernando-Momblona X, da Silva-Diz V, Munoz P (2011). The intestinal stem cell signature identifies colorectal cancer stem cells and predicts disease relapse. Cell Stem Cell.

