# Supplementary figures and images for: Salinomycin inhibits metastatic colorectal cancer growth and interferes with Wnt/β-catenin signaling in CD133+ human colorectal cancer cells
Source: BMC Cancer. 2016 Nov 17;16:896. doi: 10.1186/s12885-016-2879-8 (PMC5114842; doi:10.1186/s12885-016-2879-8)

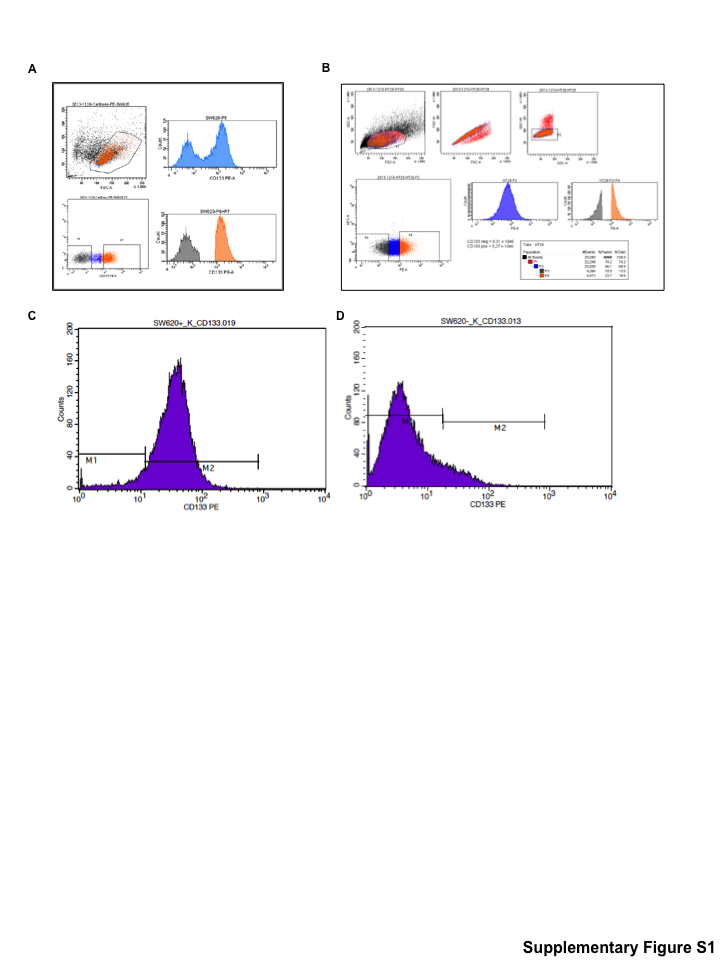

Supplement: Additional file 1: Figure S1. — Sorting of CD133+ and CD133- human colorectal cancer cells. SW620 and HT29 cells were stained with a Phycoerythyrin-conjugated CD133 antibody. Signal enhancement was performed by a two-step FASER procedure. (A + B) Representative setups before sort of CD133+ and CD133- SW620 and HT29 cells. (C + D) The purity of CD133+/CD133- cells was analyzed before the experiments were performed. (TIFF 2702 kb) [file 12885_2016_2879_MOESM1_ESM.tiff]

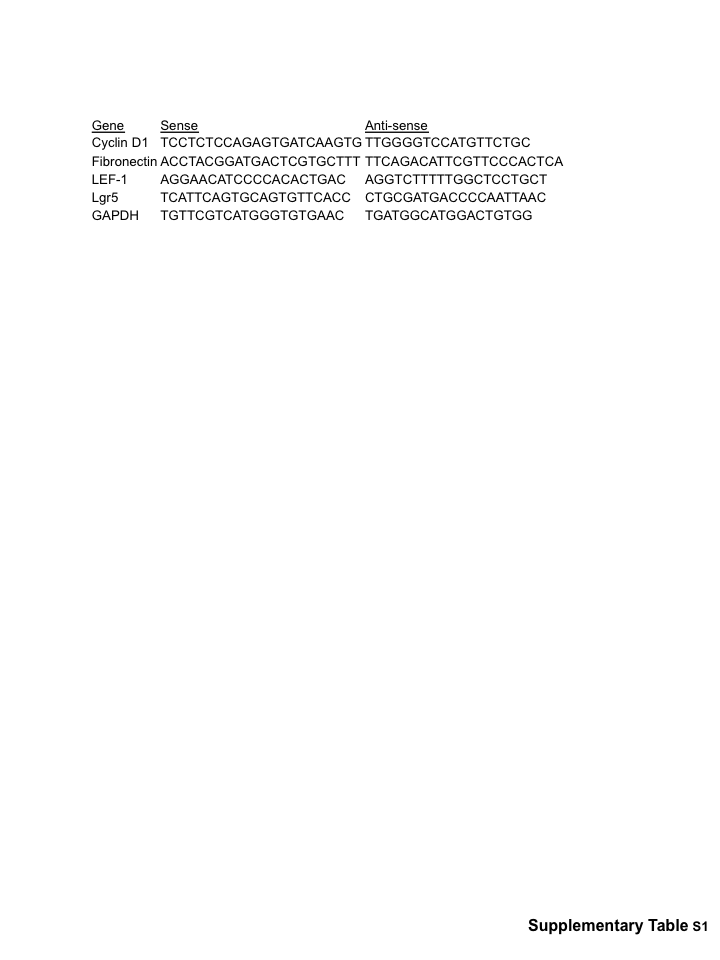

Supplement: Additional file 2: Table S1. — Sense and anti-sense primer sequences of Wnt target genes and housekeeping gene used for quantitative PCR analysis. (TIFF 2702 kb) [file 12885_2016_2879_MOESM2_ESM.tiff]

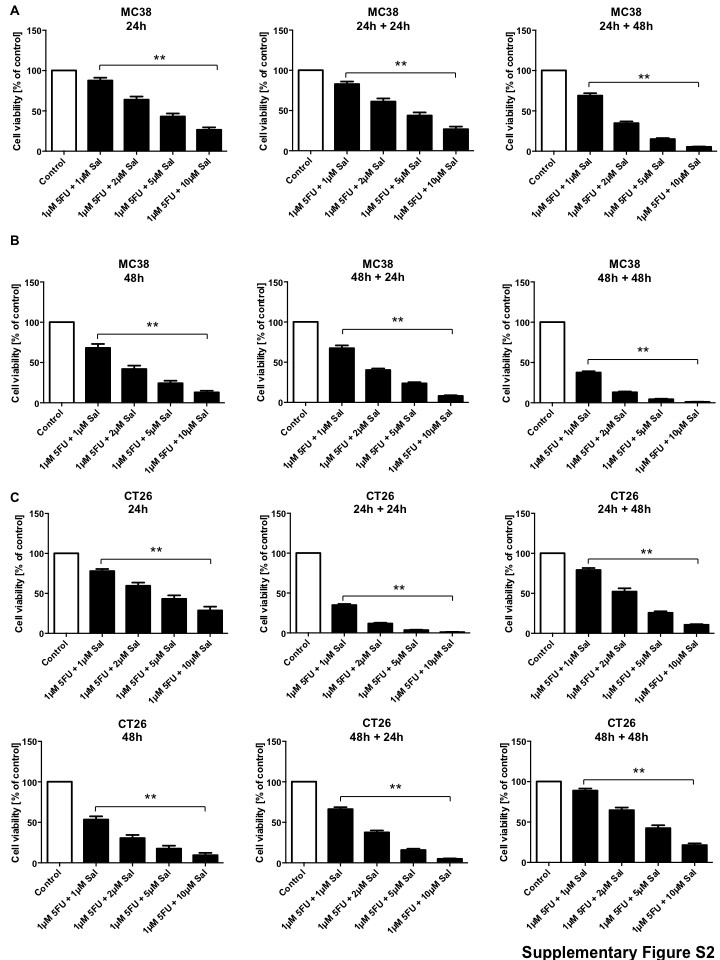

Supplement: Additional file 3: Figure S2. — The impact of combined treatment with Salinomycin and 5-FU on murine CRC cell proliferation. The effect of combined treatment with Salinomycin and 5-FU on tumor cell proliferation was performed using the WST-1 assay. Therefore, 5 × 10^3^ MC38 (A + B) and CT26 (C + D) cells were cultured in microtitre plates in the absence or presence of Salinomycin (1 μM, 2 μM, 5 μM and 10 μM) and 1 μM 5-FU. Treatment was performed either for 24 h and 48 h or for another 24–48 h with fresh medium. Results are displayed as summary of at least 3 independent experiments as mean ± SD; * p < 0.05 and ** p < 0.001 compared with control. (TIFF 2702 kb) [file 12885_2016_2879_MOESM3_ESM.tiff]

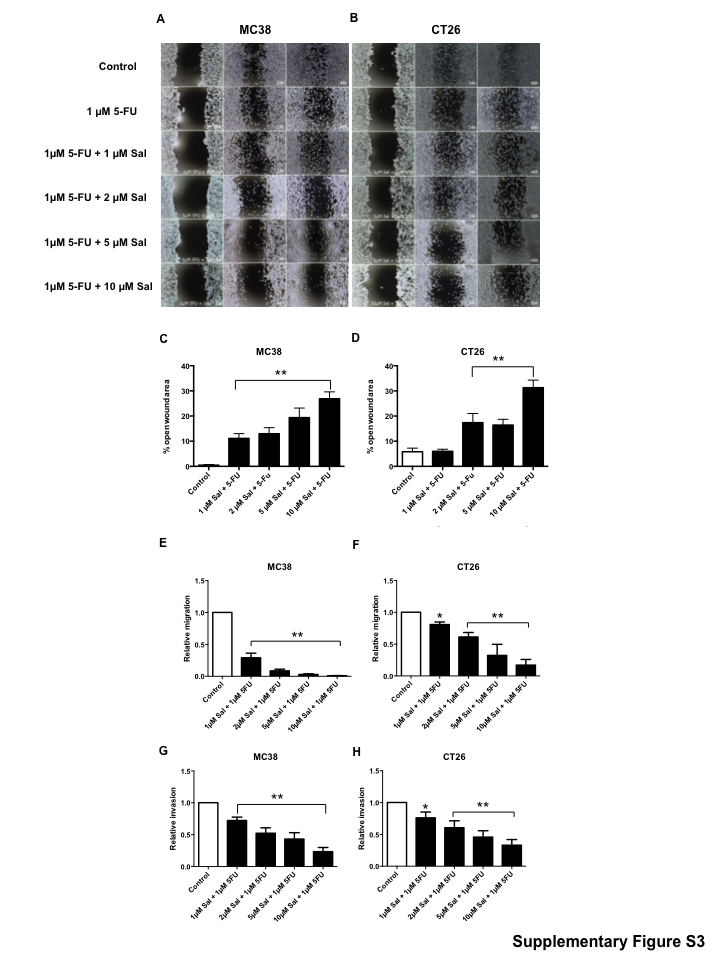

Supplement: Additional file 4: Figure S3. — Combined treatment with Salinomycin and 5-FU reduces migration and invasion of murine CRC cells and induces necrotic cell death. To visualize tumor cell migration 0.5 × 10^6^ MC38 (A) and CT26 (B) cells were cultured in 6-well plates until confluence. A scratch was created in the middle of the monolayer and cells were treated with Salinomycin (1 μM, 2 μM, 5 μM and 10 μM) and 1 μM 5-FU. Cell migration was assessed by phase-contrast microscopy and images were captured at the beginning of treatment and after 24 and 48 h at a magnification of 100. The open wound area after 48 h in cultured MC38 (C) and CT26 cells (D) was calculated and displayed as a summary of 3 independent experiments as mean ± SD; ** p < 0.001 compared with control. For transwell-analysis of tumor cell migration 1 × 10^5^ MC38 or CT26 cells were seeded in 6-well plates equipped with a transwell insert and exposed to Salinomycin (1 μM, 2 μM, 5 μM and 10 μM) and 1 μM 5-FU. After 48 h membranes were stained with crystal violet solution and migrated cells were isolated from the lower side of the membrane and quantified by ELISA reader (E + F). Alternatively, MC38 and CT26 were cultured in Matrigel-coated transwell inserts. After 48 h or further incubation for another 48 h with fresh culture medium the number of invasive migrated cells was quantified as described above (G + H). Results are shown as representative images of stained membranes at a magnification of 100 or as summary of at least 3 independent experiments as mean ± SD; * p < 0.05 and ** p < 0.001 compared with control. For detection of cell death 0.5 × 10^6^ MC38 (I) or CT26 (J) cells were seeded in 6-well plates and grown until confluence following exposure to Salinomycin (1, 2, 5 and 10 μM) and 1 μM 5-FU for 24 h. Detection of cell death was performed using AnnexinV-FITC and PI staining and cells analyzed by flowcytometry. Results are displayed as representative dot blots or as summary of at least 3 independent experiments; ** p < 0.001 compared w [file 12885_2016_2879_MOESM4_ESM.zip › Supplementary Figure S3 A-HR5- I-JR5/Supplementary Figure S3 A-HR5.tiff]

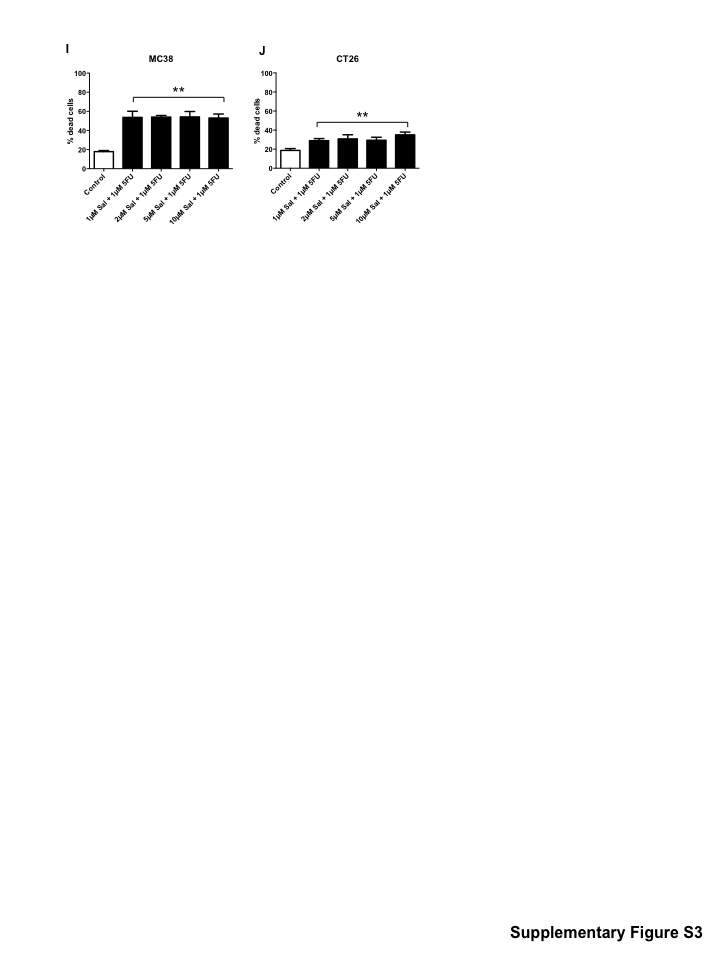

Supplement: Additional file 4: Figure S3. — Combined treatment with Salinomycin and 5-FU reduces migration and invasion of murine CRC cells and induces necrotic cell death. To visualize tumor cell migration 0.5 × 10^6^ MC38 (A) and CT26 (B) cells were cultured in 6-well plates until confluence. A scratch was created in the middle of the monolayer and cells were treated with Salinomycin (1 μM, 2 μM, 5 μM and 10 μM) and 1 μM 5-FU. Cell migration was assessed by phase-contrast microscopy and images were captured at the beginning of treatment and after 24 and 48 h at a magnification of 100. The open wound area after 48 h in cultured MC38 (C) and CT26 cells (D) was calculated and displayed as a summary of 3 independent experiments as mean ± SD; ** p < 0.001 compared with control. For transwell-analysis of tumor cell migration 1 × 10^5^ MC38 or CT26 cells were seeded in 6-well plates equipped with a transwell insert and exposed to Salinomycin (1 μM, 2 μM, 5 μM and 10 μM) and 1 μM 5-FU. After 48 h membranes were stained with crystal violet solution and migrated cells were isolated from the lower side of the membrane and quantified by ELISA reader (E + F). Alternatively, MC38 and CT26 were cultured in Matrigel-coated transwell inserts. After 48 h or further incubation for another 48 h with fresh culture medium the number of invasive migrated cells was quantified as described above (G + H). Results are shown as representative images of stained membranes at a magnification of 100 or as summary of at least 3 independent experiments as mean ± SD; * p < 0.05 and ** p < 0.001 compared with control. For detection of cell death 0.5 × 10^6^ MC38 (I) or CT26 (J) cells were seeded in 6-well plates and grown until confluence following exposure to Salinomycin (1, 2, 5 and 10 μM) and 1 μM 5-FU for 24 h. Detection of cell death was performed using AnnexinV-FITC and PI staining and cells analyzed by flowcytometry. Results are displayed as representative dot blots or as summary of at least 3 independent experiments; ** p < 0.001 compared w [file 12885_2016_2879_MOESM4_ESM.zip › Supplementary Figure S3 A-HR5- I-JR5/Supplementary Figure S3 I-JR5.tiff]

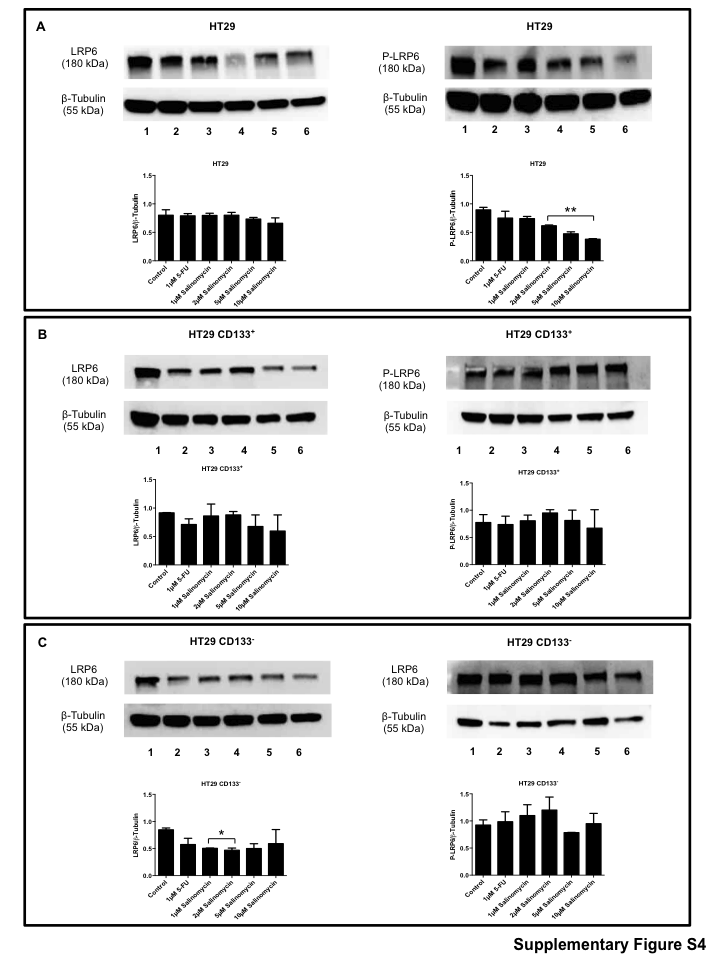

Supplement: Additional file 5: Figure S4. — Salinomycin interferes with Wnt signaling in HT20 CD133+/- cells. The impact of Salinomycin treatment on the Wnt/β-catenin signaling pathway in HT29 CD133+/CD233- cells was investigated. For protein expression analysis 1 × 10^6^ total HT29 (A), HT29-CD133+ (B) or HT29-CD133- (C) cells were cultured in 6-well plates in the absence or presence of 1 μM 5-FU or increasing concentrations of Salinomycin (1 μM, 2 μM, 5 μM and 10 μM). After protein extraction western blotting was performed using specific antibodies against total LRP6 and phosphorylated (P-LRP6). (TIFF 2702 kb) [file 12885_2016_2879_MOESM5_ESM.tiff]

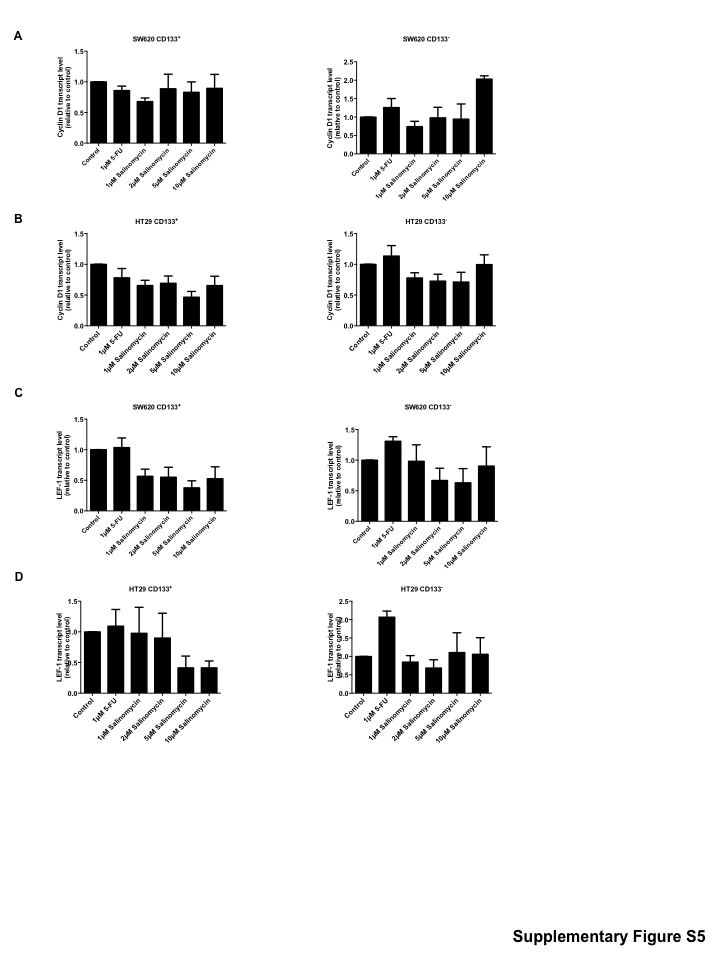

Supplement: Additional file 6: Figure S5. — mRNA expression pattern of Cyclin D1 and LEF1 after treatment with Salinomycin. The mRNA expression levels of Cyclin D1 (A + B) and LEF1 (C + D) in CD133+CD133- SW620 and CD133+/CD133- HT29 cells were measured by quantitative PCR after treatment with1 μM 5-FU or increasing concentrations of Salinomycin (1 μM, 2 μM, 5 μM and 10 μM). (TIFF 2702 kb) [file 12885_2016_2879_MOESM6_ESM.tiff]

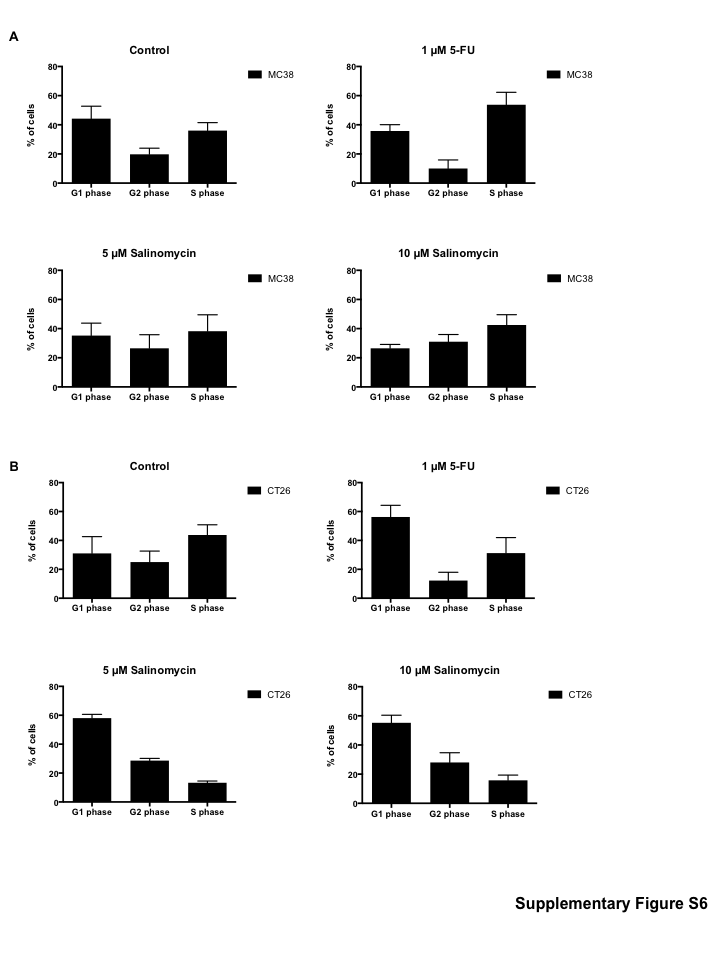

Supplement: Additional file 7: Figure S6. — Salinomycin influences the cell cycle of murine CRC cells. For cell cycle analysis 1 × 10^3^ MC38 or CT26 cells were seeded in 6-well plates and grown until confluence and then further cultured with medium, 1 μM FU and 5 or 10 μM Salinomycin for 24 h. After the end of treatment, cells were stained with PI and analyzed by flowcytometry. Sal-treatment resulted in a accumulation in the G2 phase in MC38 cells and accumulation in the G1 phase in CT26 cells, respectively. Results are summarized of at least 3 independent experiments. (TIFF 2702 kb) [file 12885_2016_2879_MOESM7_ESM.tiff]
